# Autophagy in Zika Virus Infection: A Possible Therapeutic Target to Counteract Viral Replication

**DOI:** 10.3390/ijms20051048

**Published:** 2019-02-28

**Authors:** Rossella Gratton, Almerinda Agrelli, Paola Maura Tricarico, Lucas Brandão, Sergio Crovella

**Affiliations:** 1 Department of Advanced Diagnostics, IRCCS Burlo Garofolo, Via dell’Istria 65/1, 34137 Trieste, Italy; tricaricopa@gmail.com; 2 Laboratory of Immunopathology Keizo Asami (LIKA), Federal University of Pernambuco, Av. Prof. Moraes Rego, 1235—Cidade Universitária, 50670-901 Recife, Brazil; almerindaapimentel@gmail.com; 3 Department of Pathology, Federal University of Pernambuco, Av. Prof. Moraes Rego, 1235—Cidade Universitária, 50670-901 Recife, Brazil; dpatologia@ufpe.br; 4 Department of Medical Surgical and Health Sciences, University of Trieste, Strada di Fiume 447, 34149 Trieste, Italy; sergio.crovella@burlo.trieste.it

**Keywords:** Zika virus, autophagy, Akt-mTOR, innate immunity, therapeutic targets

## Abstract

Zika virus (ZIKV) still constitutes a public health concern, however, no vaccines or therapies are currently approved for treatment. A fundamental process involved in ZIKV infection is autophagy, a cellular catabolic pathway delivering cytoplasmic cargo to the lysosome for degradation—considered as a primordial form of innate immunity against invading microorganisms. ZIKV is thought to inhibit the Akt-mTOR signaling pathway, which causes aberrant activation of autophagy promoting viral replication and propagation. It is therefore appealing to study the role of autophagic molecular effectors during viral infection to identify potential targets for anti-ZIKV therapeutic intervention.

## 1. Introduction

Zika virus (ZIKV) is an arthropod-borne virus (arbovirus), member of the *Flaviviridae* family, first isolated in 1947 from a febrile rhesus macaque monkey in the Zika Forest (Uganda). Symptomatic ZIKV infections have always been limited to small clusters of patients and to sporadic cases causing localized outbreaks throughout Africa, Asia, and Oceania. Nevertheless, this emerging arbovirus infection reached a global scale of diffusion since its arrival in the Americas in 2015, particularly devastating in the northeastern territories of Brazil. This virus was declared a public health emergency after reports of clinical complications such as neonatal microcephaly and other neurological disorders following infection [1,2]. Even if viral circulation is currently at low levels in endemic areas, presumably due to the development of herd immunity, Zika is still considered an ongoing challenge requiring strict surveillance [3]. Epidemiological data from Brazilian territories in 2018 provide support to the notion that the impact of ZIKV infection on health is diminishing. Indeed, the Brazilian Ministry of Health asserted that of the 16,735 suspected changes in the growth and development of children exposed to ZIKV infection, 3,267 (19.5%) were confirmed cases [4]. In spite many efforts over the last years aimed at developing preventive vaccines and strategies for limiting the effects of ZIKV infection, few results have been obtained so far. Therefore, a characterization of ZIKV biology is required in order to identify potential targets for effective therapeutic interventions [3]. 

**Figure 1:**
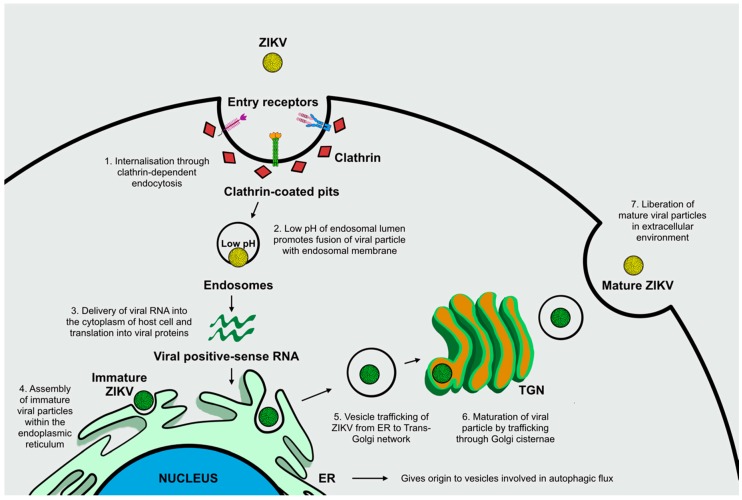
Mechanisms involved in Zika virus-host cell interactions. The binding of ZIKV structural glycoproteins to cellular entry receptors triggers viral internalization through clathrin-dependent endocytosis (**1**). The endosomal lumen’s acidic pH induces conformational changes of viral surface glycoproteins thus allowing the fusion of viral envelope with endosomal membrane, causing the release of viral RNA into the cytosol (**2**). Viral RNA is then translated into viral proteins (**3**). Immature viral particles assemble within the endoplasmic reticulum (ER) (**4**), and vesicle trafficking enables the transition of ZIKV from the ER to the Golgi network (**5**). ZIKV then passes through the cisternae of the Golgi apparatus and promotes viral maturation (**6**). Mature ZIKV particles are delivered and liberated into the extracellular environment (**7**).

ZIKV, a 50 nm enveloped and icosahedral particle with an 11 kb positive-strand RNA genome, is typically transmitted by bites from infected *Aedes* genus mosquitoes, in addition to mother-to-child transmission, blood transfusion, sexual contact, and organ transplantation [2]. ZIKV’s viral RNA genome encodes three structural proteins (membrane precursor prM, envelope E glycoprotein, and core C) and seven non-structural (NS) proteins (NS1, NS2A, NS2B, NS3, NS4A, NS4B, and NS5), which have been found to play a pivotal role in assisting viral pathogenesis and propagation by mediating viral entry and promoting viral translation and replication [5,6]. Viral structural glycoproteins, in particular envelope E glycoprotein, mediate binding to cellular receptors, thereby triggering endocytotic pathways. These interactions between cellular receptors and glycoproteins allow ZIKV to infect specific cellular types including fibroblasts, immature dendritic cells, epidermal keratinocytes, and stem-cell-derived human neural progenitor cells [7]. The uptake of viral particles occurs primarily through clathrin-dependent endocytosis. Surface glycoproteins of internalized viral particles undergo conformational changes due to the endosomal lumen’s acidic environment, which promote viral envelope fusion with the endosomal membrane. This completes the entry process, which implies the delivery of viral RNA into the cytoplasm of the host cell. The positive-sense RNA is translated into a polyprotein, which is subsequently cleaved to release structural and NS proteins [7]. Cellular compartments such as the endoplasmic reticulum (ER) and the Golgi apparatus, seem to be crucial for viral replication and propagation. First, ER membranes give rise to the vesicles involved in autophagic flux, a cellular mechanism exploited and manipulated by *Flaviviruses* in order to enhance their own replication and initiate infection [8]. Second, immature viral particles are assembled within the ER and virions traffic through the Golgi network for particle maturation prior to the release from the infected cell. Mature particles are then delivered into the extracellular environment where they are ready to commence a new infectious life cycle (Figure 1) [7,9].

The goal of this review is to investigate in depth the role of autophagy during ZIKV infection. The precise molecular mechanisms involved in ZIKV-induced autophagy have not been fully elucidated. Nevertheless, previous findings strongly suggest that a greater insight and description of the effectors participating in these cellular processes might be required for highlighting potential molecules to be used as possible therapeutic targets, and to design compounds able to modulate the activity of autophagy in ZIKV-infected cells [8,10].

## 2. Autophagy

Autophagy is a strictly regulated cellular degradative pathway involving the delivery of cytoplasmic cargoes for lysosomal degradation and subsequent recycling of the resulting macromolecular constituents. Currently three forms of autophagy have been identified and well characterized—microautophagy, chaperone-mediated autophagy, and macroautophagy—that differ in their type of exploited physiological function and mode of cargo delivery to lysosomes [11]. In this review, we focus on macroautophagy (herein referred to as autophagy), a mechanism by which cell substrates are included in double-membrane vesicles termed autophagosomes [12]. Eukaryotic cells benefit from autophagy, which is the process involved in removing damaged or long-living organelles and redundant proteins (including peroxisomes, mitochondria, and ER) through lysosome machinery. Indeed, this cellular process is known to occur virtually at basal levels in every living cell in the organism, but it can be rapidly upregulated to respond to unfavorable stress signals by generating energy for cellular homeostasis maintenance [11].

The initial steps include phagophore or pre-autophagosome formation, an isolation membrane able to enclose and fuse its edges forming an autophagosome, which is a double-membrane partition that includes cytoplasmic material. Autophagosomes then fuse with lysosomes forming autolysosome or degradative autophagosomes, compartments where sequestered intra-autophagosomal components are degraded by lysosomal hydroxilases [11].

Several factors and regulatory kinases can mediate the activation and progression of this process. The main regulatory molecules are called autophagy-related proteins (Atgs), the majority of which are highly conserved from yeast to humans with many yeast orthologs found in mammalian cells. 

1A/1B light-chain 3 (LC3) is an Atg protein that plays a central role in autophagy. During the engulfment of cytosolic components by the autophagosomes, a soluble and cytosolic form of LC3 (LC3-I) is lipidated in conjugation with phosphatidylethanolamine into an active moiety of LC3 (LC3-II). This form goes to autophagosomal membranes promoting membrane elongation. Following fusion of autophagosomes and lysosomes, LC3-II is still retained in the membrane and is itself degraded with autophagosomal components by lysosome hydroxilases. Therefore, in the presence of stimuli cable of enhancing autophagic responses, there is an increment in LC3 cellular levels while a correct completion of the process is characterized by its reduction [13,14].

LC3-II interacts with a class of autophagy adaptors known as sequestosome-1/p62-like receptors (SLRs), which are pathogen recognition receptors (PRRs) cable of selectively targeting diverse microorganisms towards selective autophagic degradation. Typically, SLRs contain three principal domains: cargo recognition and capture domains such as ubiquitin-associate (UBA) domain allowing a strong and selective binding to ubiquitin chains; LC3-interacting region (LIR), through which SLRs recognize multiple sites of autophagosome-localized LC3; and additional protein interaction domains involved in inflammatory processes. In response to bacterial and viral infections the main SLRs are given by sequestosome-1/p62 (p62), autophagy cargo receptor (NBR1), and calcium-binding and coiled-coil domain-containing protein 2 (CALCOCO2). These proteins may lead to selective autophagy mainly via ubiquitin-signaling of invasive microorganisms. SLRs are able to pack ubiquinated microbial components via the UBA site, while the interaction with LC3 through the LIR region promotes the delivery of packed substrates towards the autophagic route. Therefore, SLRs constitutes a molecular bridge between LC3 and ubiquinated cargoes, thereby promoting the enclosure and degradation into autophagosomes of target molecules [15,16]. 

### 2.1. Autophagy and Its Role in the Immune Responses Against Invading Microorganisms

A strongly emergent concept correlates autophagy with immune functions. This cellular process can be considered a primordial form of innate immunity counteracting invading microorganisms. In mammalian cells these primordial functions of autophagy have evolved, and are now integrated in several innate and adaptive pathways. Indeed, autophagy has been seen to mediate multiple aspects of the immune response including direct elimination of intracellular pathogens comprising viruses, the control of adaptive responses through the promotion of antigen presentation, and the control of inflammation and inflammasome activation with subsequent cytokine secretion [17]. 

**Figure 2:**
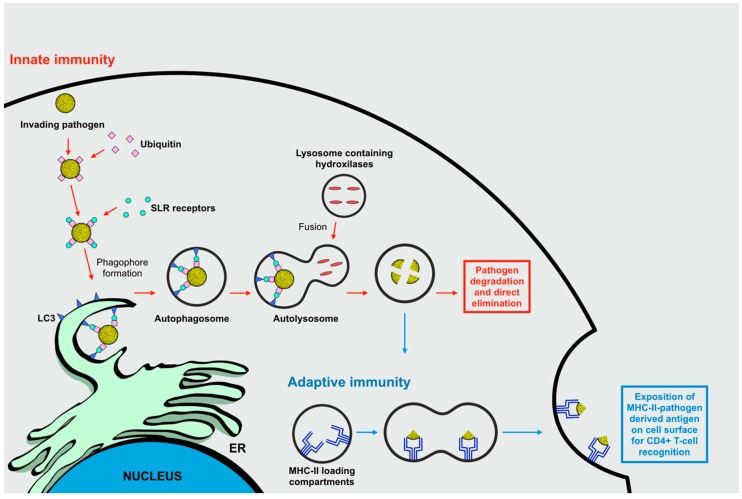
The role of autophagy during innate and adaptive immune responses against invading microorganisms. As a fundamental component of the innate immune response, selective autophagy degrades invading pathogens principally through ubiquitin-signaling. Ubiquinated pathogens are recognized by sequestosome-1/p62-like receptors (SLRs), which are involved in creating a molecular bridge between 1A/1B light-chain 3 (LC3) recruited on ER membranes and the ubiquinated microbial components, thus promoting their enclosure into the autophagosomes of target molecules. The subsequent fusion of autophagosomes with lysosomes leads to pathogen degradation and direct elimination. These vesicles containing exogenous antigenic peptides, derived from lysosome degradation, fuse with major histocompatibility complex class II (MHC-II) loading-compartments. After loading on MHC-II, the peptides are transported to the cell surface in order to induce the stimulation of a CD4^+^ T-cell anti-viral adaptive immune response.

During viral infections, innate immunity disposes of several defense mechanisms that can rapidly detect the presence of invading microorganisms, and are promptly solicited to counteract invasion by mounting an efficient antiviral first-line response. The principle defense mechanism is given via the recognition of viral-conserved pathogen-associated molecular patterns (PAMPs) by cytosolic or membrane-bound PRRs. This is followed by the transduction of intracellular signals culminating in the stimulation of innate immune responses, principally including the activation of transcription factors that regulate the synthesis of inflammatory cytokines. During the progressive mounting of this innate response, autophagy constitutes an integrated, valid, and immediate option by exerting a direct immune function. Indeed, autophagy is a constantly ongoing cellular process that traps pathogens and mediates their prompt degradation following infection by capturing intracellular pathogens into autophagosomes (Figure 2) [17,18]. 

Nevertheless, autophagic machinery activation is not limited to the progression of innate immune responses, it also performs a crucial role in the context of adaptive immunity during viral infections by influencing cytosolic antigen processing and presentation for major histocompatibility complex class II (MHC-II) molecules. MHC-II molecules present exogenous antigenic peptides derived from lysosome degradation. After loading on MHC-II, the peptides are transported to the cell surface in order to induce a CD4^+^ T-cell response (Figure 2) [16]. Interestingly, autophagosomes can deliver pathogen-derived antigens to MHC-II loading compartments as demonstrated by morphological analysis that showed a 30%–50% co-staining between MHC-II compartments and the autophagosome marker LC3 [19].

In addition, a complex association has been found between autophagy and the regulation of inflammatory responses, affecting the secretion of inflammatory and anti-microbial mediators, and inflammasome activation. Inflammation constitutes a vital host response to tissue loss and cellular homeostasis, thus promoting host defense, tissue repair and remodeling, and metabolism regulation. During infection, a cascade of signals leads to the recruitment of inflammatory cells (neutrophils and macrophages), which phagocytose damaged cells and infectious agents, thus promoting the secretion of pro-inflammatory cytokines and chemokines (e.g., tumor necrosis factor (TNF) and interleukin 1 (IL-1)) for the subsequent activation of adaptive immune responses [17,18]. 

Different PRRs can recognize viral PAMPs, thus detecting the initiation of a viral infection. Following recognition, the activation of signaling pathways through different classes of PRRs (toll-like receptors (TLRs), nucleotide oligomerization domain-like receptors (NLRs), and RIG-I-like receptors (RLRs)), have been shown to converge with the autophagic route [17].

The principle PRRs involved in the recognition of viral PAMPs are TLRs, located on the plasma membrane (TLR1/2/4/5/6/10) for recognition of microbial membrane components or inside endosomal compartments (TLR 3/7/8/9) where uptaken microbial partitions are directed. Signal transduction is primarily mediated by toll/interleukin-1 receptor (TIR)-domain-containing adapter-inducing interferon-β (TRIF) or myeloid differentiation primary response gene 88 (Myd88), for the activation of transcription factors including NF-kB, IRF3/7, and API-1, which induce the expression of pro-inflammatory cytokines (e.g., IL-6 and IL-1 β) and type 1 interferons (INF), thus leading to the modulation of anti-viral immune responses [17,20,21,22]. TLR signaling pathways are thought to induce autophagy principally by promoting autophagosome formation, presumably in a Myd88 or TRIF-dependent manner [23]. Furthermore, autophagy is known to enhance viral recognition and modulate downstream synthesis of inflammatory cytokine production principally by delivering viral PAMPs to their receptors [24]. 

Another crucial connection between inflammatory responses and autophagy is related to the regulation of inflammasome activation [20]. Indeed, autophagy is known to regulate this activation by controlling the secretion levels of pro-inflammatory cytokines including IL-18 and IL-1β, though the mechanisms underlining this regulatory activity are still under debate [20,25]. 

**Figure 3:**
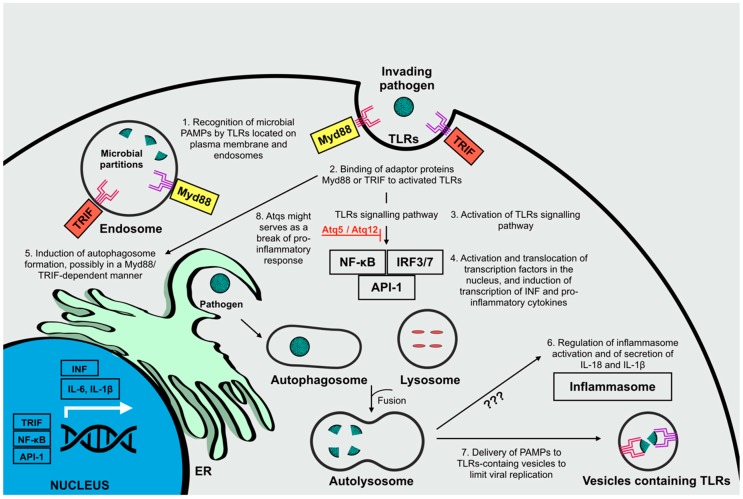
The role of autophagy in the regulation of inflammatory responses against invading pathogens. Microbial pathogen-associated molecular patterns (PAMPs) are recognized by toll-like receptors (TLRs) located on the plasma membrane or in endosomal compartments (**1**). The recognition of activated TLRs by adaptor proteins Myd88 or TRIF (**2**), activates TLR signaling pathways (**3**), involved in the activation of transcription factors Nf-KB, IRF3/7, and API-1 that then translocate to the nucleus for the induction of INF and pro-inflammatory cytokines transcription (**4**). The promotion of autophagosome formation is induced in a Myd88- or TRIF-dependent manner (**5**), and leads to the regulation of inflammasome activation and secretion of pro-inflammatory cytokines, through mechanisms that remain not completely understood (**6**), and to the delivery of PAMPs to TLR-containing vesicles to limit viral replication (**7**). Besides promoting pro-inflammatory activities, autophagy also serves as an impediment on the magnitude of the host’s antiviral reaction. Indeed, autophagy-related proteins (Atg12 and Atg5) might serve as a break for the pro-inflammatory response induce by TLRs (**8**).

Nevertheless, autophagy might not only serve to sustain an inflammatory antiviral response, but also serve as an impediment on the magnitude of the host’s antiviral reaction. Recently, some Atgs (namely Atg5 and Atg12) have been shown to down-regulate some PRRs, where autophagy might also serve as a brake on the pro-inflammatory response induced by the activation of NLRs (Figure 3) [21,26,27,28]. Presumably, the role of autophagy in suppressing inflammatory responses is linked to the necessity to dampen immune signaling during viral infections to avoid excessive and potentially harming inflammatory responses. All this considered, while analyzing the interplay between inflammation and autophagy during viral infections, it is necessary to consider the possible dual function of the catabolic route: cooperation between autophagy and innate immunity, and the suppression of innate immunity by autophagy [21]. 

### 2.2. Regulation of Autophagic Flux

Autophagy is sensitive to a great variety of stimuli that are transduced by different cellular effectors. In the presence of unfavorable stress conditions, the activation of autophagic flux assures energy supplies and molecular building blocks to sustain cellular proliferation and survival [29]. Specifically, the induction of autophagy is caused by many stress-inducing factors including starvation, growth-factor withdrawal, ER stress, redox stress, hypoxia, danger-associated molecular patterns, mitochondrial damage, chemicals, and irradiation [16,30]. Viruses can trigger many of these stresses in the host cell during different stages of their replication cycle. Viral binding to target receptors, as well as viral intracellular replication, are the best described events during viral infection known to elicit stress responses and subsequent stimulation of autophagy [16]. 

However, all these signaling molecules transduce signals to a common target, the kinase mTOR, found to be directly upstream of the machinery involved in autophagosome formation. mTOR is a serine/threonine kinase considered a master regulator and gatekeeper of the autophagic pathway. In eukaryotic cells, mTOR forms two different signaling complexes: multiprotein complex 1 of mammalian target of rapamycin (mTORC1) and multiprotein complex 2 of mammalian target of rapamycin (mTORC2), composed of mTOR and multiple binding proteins. The two kinase complexes bind to diverse and distinct substrates and therefore promote the transduction of different downstream signaling pathways to modulate distinct cellular events [29,31]. 

Another aspect of autophagy subjected to severe regulation is the mobilization of membrane structures during the generation of autophagic compartments moving through the endo/exocytotic vesicular intracellular trafficking, which requires control by different GTPases [31].

Indeed, several small GTPases are indicated as crucial Atg proteins and they comprehend Rheb, Rabs, and RalB. Ras homology enriched in brain (Rheb) is known to activate, by direct and selective binding, mTORC1 [32]. Ras-related protein Rabs (Rabs) are involved in the regulation of autophagy intracellular vesicular trafficking including vesicle budding, delivery, tethering, and fusion [33]. Ras-related protein RalB (RalB) affects isolation of the pre-autophagosomal membrane and the maturation of autophagosomes by localizing on nascent autophagosomes following activation due to starvation and binding to Exo84 effector [34].

mTORC1 promotes anabolic cellular metabolism to supply the necessary building blocks for cell growth and proliferation. The activation of mTORC1 by nutrients and growth factors induces a blockade of autophagy at both a post-translation and transcriptional level through the phosphorylation of various Atgs involved in the promotion of autophagy initiation and autophagosome formation [29]. 

A well-known upstream regulator of mTORC1 is the PI3K/Akt pathway. Signaling route activation occurs at the cell membrane level following the binding of tyrosine kinase receptors (including epidermal growth factor receptor, insulin-like growth factor-1 receptor, G-protein-coupled receptors) to their ligands, causing stimulation of the PI3K/Akt signaling axis. Once activated, the PI3K catalyzes the phosphorylation of phosphoinositides to generate activated molecules—phosphatidylinositol-3,4,5-triphosphate and phosphatidylinositol-3,4-bisphosphate. The activated phosphoinositides bind and activate both the serine/threonine kinase Akt and the 3’-phosphoinositide-dependent kinase 1 (PDK-1). Next, PDK-1 phosphorylates Akt, which is then able to propagate its cellular signals to affect cell cycle progression, transcription, and apoptosis. In this context, Akt might trigger mTORC1 activation directly, catalyzing mTORC1 phosphorylation on S2448, or indirectly by phosphorylating and inactivating tuberous sclerosis complex 2 (TSC2), normally exerting an inhibitory activity on mTORC1 when bound to Rheb [35]. Once activated, mTORC1 phosphorylates and inactivates multiple Atgs such as ULK1 and Atg13, two components of the ULK complex, in addition to AMBRA1 and Atg14L, constituent of the VPS34 complex, thus preventing autophagy initiation and transcription of lysosome and autophagy genes [29]. 

On the contrary, autophagy initiation induced by stress stimuli is characterized by the inactivation of mTORC1 until the complex in reactivated by energy supplies released as products of autolysosomal degradation at the end of autophagic flux [29]. 

## 3. *Flavivirus* Infection and Autophagy

As a fundamental part of the immune system, autophagy plays a crucial role in sensing the presence of viruses and mediating their elimination. It is thought that the consumption of intracellular nutrients caused by a competition between the invading virus and the host cells’ metabolism constitutes one of the principal danger signals in the eukaryotic cell used to recognize infection and mount an appropriate antiviral immune response through the activation of autophagy [17]. Therefore, it is not surprising that several viruses have evolved mechanisms to exploit and subvert autophagic machinery, directly or indirectly, to promote their replication and propagation [8].

Describing the role of autophagy in viral infection has been elucidating the puzzle relative to *Flavivirus* pathogenesis. Indeed, autophagy provides an adequate platform for *Flavivirus* replication during the early steps of infection, allowing apoptosis inhibition, innate immunity evasion, and lipid metabolism reordering to support robust replication [36].

Apart from exerting a degradative function through the recycling of damaged or long-living organelles, redundant proteins, or invading microbes via lysosomal machinery, autophagy has been also described to possess different biogenesis-associated functions [37]. This unconventional mechanism is known as secretory autophagy, involved in exporting a great variety of cytoplasmic substrates including cytokines, contents of intracellular organelles and invading microorganisms. The study of this process seems promising for a better characterization of *Flavivirus*–host cell interactions [37,38]. 

In fact, *Flaviviruses* can hijack the autophagic response in order to enhance their own replication and facilitate the establishment of infection [8]. Therefore, unraveling the role of autophagy during *Flavivirus* infection might have implications for better understanding viral pathogenesis and aid the identification of potential therapeutic targets.

Although the specific molecular mechanisms involved in the modulation of autophagy by *Flaviviruses* has yet to be fully understood, literature findings relative to Japanese encephalitis virus (JEV), Usutu virus (USUV), West Nile virus (WNV), Dengue virus (DENV), and Zika virus (ZIKV) are currently available. 

Different studies suggest that JEV-induced autophagy exerts a crucial role in promoting viral replication. Indeed, the enhancement of viral progression following administration of autophagy inducers (e.g., rapamycin), and the suppression of viral growth after the administration of autophagy inhibitors (e.g., 3-methyladenine), create a strong correlation between JEV-induced alterations in the autophagy machinery and viral propagation [10,39]. JEV infection has been associated to an increment in LC3 lipidation levels and to its cytoplasmic aggregation, therefore leading to an upregulation of the autophagic pathway [10,36]. In addition, recent findings suggest that autophagic vesicles do not constitute a source of membrane compartments used by JEV to sustain its replication, but rather that viral-activated autophagy is capable of promoting the initial stages of infection, specifically the JEV entry process [36]. Moreover, the accumulation of autophagosomes is thought to enhance viral replication though the mechanisms underlining this process are not fully understood [39].

USUV belongs to the JEV serocomplex and has been recently, and rarely, detected in humans. Being closely related to JEV, the effects of USUV infection present a similar impact on autophagy when compared to JEV [40,41,42].

In the case of WNV, autophagy does not seem to play a pro-viral role, since this cellular process is supposedly not required for the activation and advancement of the viral life cycle [10]. Different experimental evidences support the presence of an upregulation of autophagy upon WNV infection, associated with an augmented lipidation and aggregation of LC3 [43,44]. Nevertheless, inhibition of autophagy by the depletion of essential autophagy Atg proteins, namely Atg5 and Atg7, is not associated to an alteration of WNV replication in infected cells therefore suggesting that a functional autophagy pathway is not required for WNV infectious particle production [10,45,46]. Taken together these findings indicate that the involvement of autophagy in WNV infection remains controversial, and further investigations are required to unravel the role of this pathway in WNV pathogenesis.

The re-emergence of DENV and ZIKV-infection indicated the necessity to characterise the principle mechanisms responsible for viral propagation in order to assess possible therapeutic interventions. In this context, autophagy appears to be a promising target. Indeed, substantial efforts to determine the major processes involved in the interaction between autophagy and viral infection are currently underway, which may result in interesting findings. 

**Figure 4:**
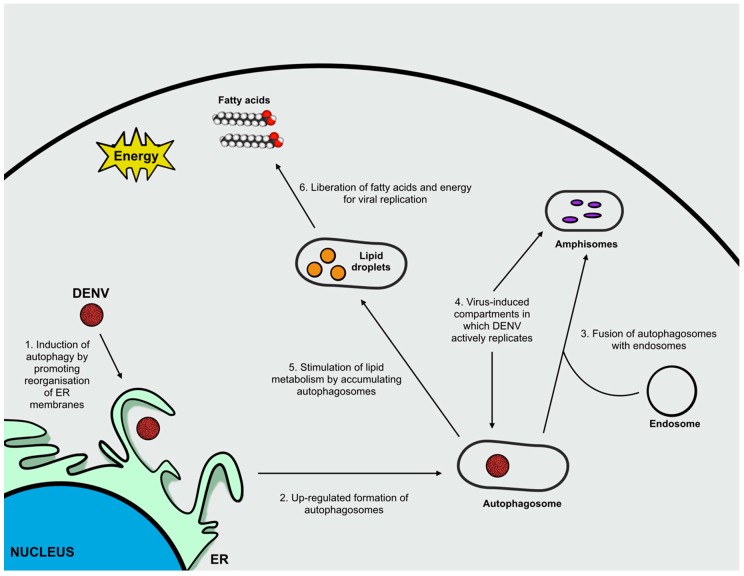
Deregulation of autophagy during Dengue virus infection. During DENV infection, autophagy has pro-viral activity. The induction of autophagy by DENV (**1**) upregulates autophagosomes formation (**2**) that fuse with endosomes to form amphisomes (**3**), virus-induced compartments in which DENV actively replicates (**4**). Accumulating autophagosomes stimulate lipid metabolism (**5**) leading to the liberation of fatty acids and energy to sustain viral replication (**6**).

DENV is known to induce autophagy and this process possesses a pro-viral activity. First, the induction of autophagy in DENV-infected cells determines membrane reorganization to form autophagosomes that fuse with endosomes to form amphisomes. In this case, both autophagosomes and amphisomes are virus-induced double-membrane compartments in which DENV actively replicates. Second, it has been hypothesized that the upregulation of autophagy might suppress the host cells’ unfolded protein response thus supporting DENV replication [8]. Finally, the induction of autophagy is crucial for targeting lipid droplets in order to stimulate lipid metabolism (lipophagy). Accumulating autophagosomes, generated following infection, preferentially associate to lipid droplets and promote the mobilization of lipid content, thus increasing lipid metabolism and β-oxidation to obtain free fatty acids and energy that are essential for sustaining viral replication [47,48]. Among the mentioned roles of autophagy in supporting DENV infection, the greatest impact of autophagy during DENV infection is associated to the promotion of lipid metabolism (Figure 4) [47]. 

**Figure 5:**
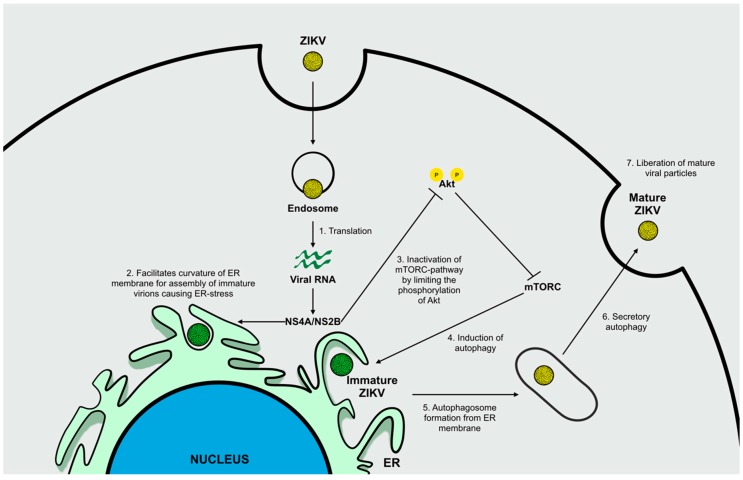
Deregulation of autophagy during ZIKV infection. Following ZIKV internalization, release of genomic material from endosomes and translation of viral RNA (**1**), NS4A and NS4B facilitate the curvature of ER membranes to promote assembly of immature viral particles (**2**) and inactivate the mTORC-pathway (**3**) by limiting Akt phosphorylation, thus inducing autophagy (**5**). The upregulation in autophagosome formation (**5**) activates secretory autophagy (**6**) with subsequent liberation of mature ZIKV particles (**7**).

Different studies have highlighted the important role of autophagy during ZIKV entry and replication after observing accumulating autophagic vesicles following viral infection in vivo and in vitro. Viral replication occurs primarily on the ER, where immature viral particles initially assemble. This stage of ZIKV infection induces ER stress, which in turn triggers autophagy activation. During this response, the non-structural proteins, NS4A and NS4B, seem to be involved in facilitating the curvature of ER membranes to generate platform sites for viral assembly, and inhibit Akt-mTORC pathway, principally by limiting Akt levels of phosphorylation, to induce autophagy and increase viral replication and release (Figure 5) [49,50,51,52].

Novel findings highlight important similarities between the mechanisms that DENV and ZIKV use in order to exploit autophagy during infection. Both DENV and ZIKV have been shown to induce the formation of LC3-containing membranes. Indeed, a common feature seems to be the requirement for LC3, which is targeted to viral-induced membranes through mechanisms other than cellular lipidation of the protein. The presence of LIR motifs in the polyproteins of ZIKV and DENV might be responsible for the viral-mediated localization of LC3 to cellular membranous compartments. Furthermore, autophagy components seem crucial for post-replication processes including viral maturation and packaging [53].

Despite these important similarities, DENV and ZIKV exploit a differential utilization of upstream components of the autophagic machinery, therefore, presumably promoting the activation of diverse upstream signaling cascades. The induction of autophagy by ZIKV appears to be linked to the activation of AMPK by phosphorylation, while DENV induces autophagy using a divergent route presumably involving the activation of VPS34 [53].

### ZIKV Infection and Autophagy

In ZIKV and other *Flavivirus*, receptor-binding is mostly mediated by the interaction of *N*-glycans conjugated to viral protein E with C-type lectin receptors (CLRs) from host-cells [54], such as DC-SIGN (Dendritic cell-specific intercellular adhesion molecule-3-grabbing non-integrin) [55,56,57,58], L-SIGN (Liver/lymph node-specific intercellular adhesion molecule-3-grabbing integrin) [58,59], MMR (Macrophage mannose receptor) [60], and CLEC5A (C-type lectin member 5A) [61]. A more recent in vitro assay demonstrated that DC-SIGN mediates ZIKV entry into cells [62]. CLRs comprise a large family of carbohydrate receptors, which bind through one or more carbohydrate recognition domains [63]. These cellular receptors recognize carbohydrate in pathogens as PAMPs and act as PRRs, interiorizing these agents into endosomes, initiating the process of antigen presentation, and elimination of the pathogen [64]. Members of this family are usually expressed in myeloid cells, including macrophages, monocytes, and dendritic cells, thus these receptors play a central role in the early steps of activating the host’s immune response [65].

Furthermore, ZIKA can acquire negatively charged lipids from the ER lumen during viral morphogenesis, mainly phosphatidylserine (PS). The PS of immature ZIKV particles misleads host cells and facilitates the infection of different tissues, including endothelial, neural, and placental [62,66,67,68,69,70]. These anionic lipids are known to bind cellular lipid receptors from two distinct families: TAM (Receptor tyrosine kinases: **T**YRO3, **A**XL and **M**ER) and TIM (T-cell immunoglobulin mucin: TIM1, TIM3 and TIM4) [71,72,73]. TIM receptors bind PS directly, whereas TAMs bind it indirectly, through a process that requires the presence of GAS6 (Growth-arrest-specific 6) or PROS (Protein S) as bridging molecules [72,74,75]. TAM receptors are expressed on the surface of macrophages, dendritic cells [76], Sertoli cells (testis) [77], retinal pigment epithelial cells [78], and neuronal cells, such as Purkinje and dentate gyrus cells [79]. In contrast, TIM receptors are mostly expressed by immune system cells, especially T cells [80,81].

The ZIKV maturation process occurs in the trans-Golgi network (TGN), where the host’s protease furin cleaves protein prM of immature particles (C, prM, and E), dissociating the pr portion from the viral particle, and releasing mature particles (C, M, and E) [82,83]. Abrogating this step leads to improper reorganization of protein E and consequent exposure of the viral membrane exhibiting prM and PS. This culminates with the release of immature viral particles exhibiting not only protein E, but also prM and PS, which could contribute to a wider range of cells susceptible to ZIKV. Additionally, a single mutation in prM was shown to be associated with the ability of ZIKV to infect cells, suggesting that prM could also act as an entry receptor [84]. Therefore, ZIKV entry into cells seems to be a process dependent on the degree of particle maturation, where multiple molecules can be explored for infectious entry dictating cell tropism.

**Figure 6:**
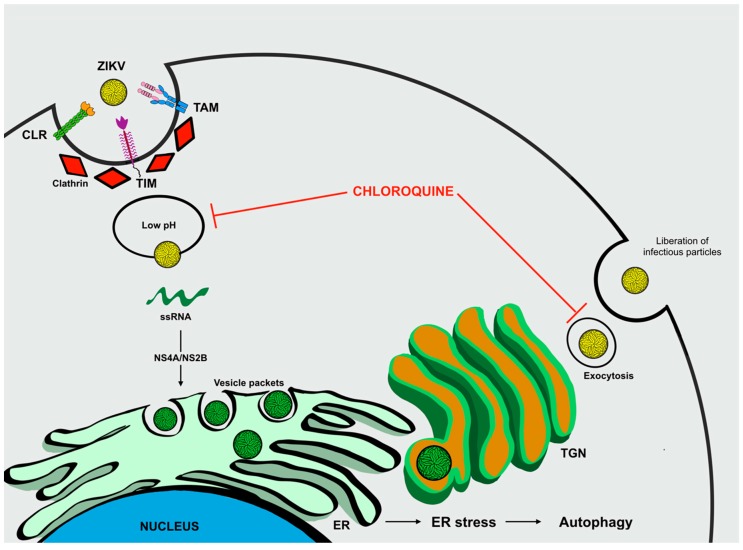
The role of autophagy in ZIKV infection. The ZIKV entry mechanism is mediated by a wide range of receptors. After membrane fusion, viral genetic material is released into the cytoplasm and translated into proteins. The formation of vesicle packets causes ER stress and triggers autophagy. Chloroquine acts by increasing the pH within the endosomes, inhibiting membrane fusion and exocytosis by secretory autophagy.

Following association with one or more host receptors, ZIKV infiltrates cells through clathrin-mediated endocytosis [85], a vesicular trafficking process that transports cargo molecules from the cell surface to its interior [86]. Initially, a pre-existing clathrin-coated pit enfolds viral particles and the invagination on the plasma membrane is closed by excision of the dynamin-mediated membrane to form a clathrin-coated vesicle. Subsequently, the endocytic vesicle carrying the virus is delivered to the initial endosomes, which mature into late endosomes [87]. Then, the acidic pH of the endosome compartment triggers a conformational change in protein E, leading to viral membrane fusion with the endosome membrane and further release of viral RNA into the cytoplasm, enabling replication and translation processes [88]. In this regard, chloroquine is thought to inhibit membrane fusion by raising the pH in the lumen of endosomes (Figure 6) [89].

Viral replication machinery is established at the ER membrane, which undergoes a structural rearrangement mediated by ZIKV structural proteins, NS4A and NS4B [90]. These invaginations inside the ER lumen are termed “vesicle packets”, and provide an ideal environment for biogenesis. The formation of these replication complexes causes ER stress, which is thought to be the main mechanism by which autophagy is triggered during ZIKV infection [49]. NS4A and NS4B have also been shown to promote autophagy in HeLa and fetal neural stem cells by inhibiting the Akt-mTOR pathway, thus increasing ZIKV replication and preventing neurogenesis [51].

During morphogenesis, the newly synthesized viral genomes are assembled into virions that bud into the ER lumen, thus the viral membrane ends up reflecting the ER membrane composition, which has PS in its luminal leaflet [91]. Then, these particles traffic through the TGN, for maturation, and further secretory pathways [82].

It is important to note that proteins lacking the N-terminal leader peptides cannot enter the ER-TGN pathway for secretion. These proteins, such as viral polypeptides, may be exported unconventionally through secretory autophagy [90]. Different from degradative autophagy, the secretory autophagy machinery causes the secretion of viral particles instead of their degradation [37]. Therefore, ZIKV may exploit secretory autophagy to facilitate the release of mature viral particles. Nevertheless, the mechanisms by which secretory autophagy branches from degradative autophagy and how ZIKV explores it, are not yet understood. On this matter, an alkalizer drug such as chloroquine may inhibit secretory autophagy and suppress degradative autophagy once acidic pH is necessary for the activation of lysosomal hydrolases.

## 4. Therapy Development and Approaches to Counteract ZIKV Infection

ZIKV is a recent emerging infectious disease characterized by its rapid spread and high virulence. Clinical manifestations following infection are generally mild, self-limited, and non-specific, which include symptoms such as fever, headache, arthralgia, myalgia, asthenia, and non-purulent conjunctivitis, found to be very similar to those caused by other arboviruses, therefore confounding the diagnosis [2]. Nevertheless, more severe outcomes have been described including the development of microcephaly in newborns, and grave neurological impairments in adults such as the Guillain Barrè syndrome (GBS) [89,92,93,94]. These complications possibly correlate with the unavailability of vaccines, therapies, or effective prophylactic strategies to counteract the infection [95]. Different ZIKV vaccine candidates are currently under development, and clinical trials entail nucleic acid based vaccines (RNA or DNA), virus-like particle, recombinant, and vectored vaccines [89,96]. Other than acetaminophen to control pain and fever, administration of fluids to prevent dehydration, and anti-histamines to prevent rushes, no specific anti-viral drugs for ZIKV have yet been identified. Recently, several compounds have been tested in order to assess a possible effective therapeutic strategy to neutralize the infection [95]. Indeed, in a scenario lacking accepted anti-ZIKV treatments, researchers are proceeding with testing repurposed available drugs already approved for use in pregnant woman, which seems to be the most effective way for reducing viral infection in adults and to limit birth defects in newborns. 

What has emerged is the necessity to investigate the genetic and molecular components underlining ZIKV biology and viral pathogenicity. Knowledge of the exact molecular processes and effectors mediating all the different stages of viral replication and host cell-virus interactions, are crucial in order to test repurposed or newly designed drugs that can be used as anti-viral agents against ZIKV.

### Targeting Autophagy to Limit ZIKV Infection: A Perspective Approach

Compound testing and drug design to block receptor binding, endosomal fusion, viral replication, and release from host cells is of contingent need to make rapid progress in the development of novel therapies that might work efficiently to fight ZIKV. 

Based on novel and crucial findings related to autophagy in promoting viral replication and progression of infection, we describe the activity of chloroquine, a compound found to efficiently limit ZIKV infection in vivo and in vitro. We also highlight other compounds known to act on autophagy, for which we suggest further testing to identify those with anti-ZIKV activity. Our intent is to highlight the importance of targeting autophagy to limit ZIKV infection.

Chloroquine (CQ) is an FDA-approved antimalarial compound with high tolerability, low cost, and immunomodulatory properties for which no substantiated reports indicate an association between drug administration and fetal harm or risk during pregnancy [97]. Due to its interesting properties, the usage of CQ has been repurposed also against other pathogens. Specifically, CQ has recently been seen to counteract ZIKV infection in different in vivo and in vitro models [50,97,98].

CQ is a 9-aminoquinolone, possessing multiple biochemical properties with direct antiviral effects. CQ is a weak base cable of reaching intracellular compartments, and is concentrated within acidic organelles including lysosomes, Golgi vesicles and endosomes. CQ acts by increasing the pH acidic organelles, therefore, disrupting the activity of several enzymes (e.g., acidic hydroxylases) and inhibiting protein post-translational modifications. It is therefore clear that CQ can disrupt the pH-dependent stages of viral replication for several members of the *Flaviviridae*, *Retroviridae,* and *Coronoviridae* families [99,100,101]. 

In the case of ZIKV infection, the acidic pH of endosome compartments is crucial for triggering conformational changes of viral glycoproteins thus allowing the fusion of the viral and endosomal membranes with subsequent release of viral RNA into the cytoplasm, enabling replication and translation processes [88]. In this context, CQ might act by inhibiting membrane fusion by raising the pH in the lumen of endosomes [89]. 

Altering the pH in the ER compartment and TGN vesicles is though to allow CQ to limit post-translational modifications of viral glycoproteins occurring in these districts since residing glycosyl-transferases and proteases require low pH [99,102]. In addition, pH increase caused by CQ, impairs the normal proteolytic processing of prM to M thus impairing viral infectivity [103].

Endosomes, ER, and TGN are cellular organelles that exert fundamental roles of ensuring a correct initiation, progression, and completion of autophagic flux. Therefore, altering the homeostasis of these compartments will inhibit the autophagic response. CQ negatively interferes in the fusion of autophagosomes with lysosomes, causing an accumulation of sequestered materials, including viral particles, either in autophagosomes or autophagolysosomes [104]. We can hypothesize that a possible mechanism through which ZIKV might exploit secretory autophagy and suppress degradative autophagy to facilitate the release of mature viral particles might reside in the alkalizer properties of CQ since acidic pH is necessary for the activation of lysosomal hydrolases.

Even though the activity of CQ seems promising in counteracting ZIKV infection, some controversial findings have been also reported. In an interesting work conducted by Adcock et al., CQ failed to inhibit ZIKV replication in their in vitro model, and showed higher cytotoxicity when compared to other CQ analogues [105]. Nevertheless, it is important to notice that CQ has been reported to decrease the number of ZIKV-infected neural cells in diverse cell models [89]. These different results highlight an important aspect to consider when testing potential anti-*Flavivirus* agents: *Flavivirus* entry and replication mechanisms are cell-specific, which might also render the effects of putative inhibitors cell-specific [105]; consequently, each compound should be tested on several host cell models prior to its consideration as a potential drug for limiting ZIKV infection.

Apart from CQ, other autophagy inhibiting lysosomotropic agents might be tested to reveal a possible anti-ZIKV activity and they include: Lys05, a bivalent aminoquinoline that causes a significant augmentation in lysosomal pH [106]; ARN5187, autophagosome maturation blocker [107]; and Quinacrine, anti-malarial drug acting as inhibitor of endosome/lysosome acidification [108].

Catalytic inhibitors of ULK kinase including MRT68921, Compound 6, and SBI-0206965, even if at the very early stages of drug discovery, seem promising in targeting the autophagic process [109,110].

Compounds that inhibit the later stages of autophagy, the degradation of autophagosome content by lysosome enzymes, might also be considered as effective agents against ZIKV which include: Pepstatin A and E64d, inhibitors of lysosomal proteases [111]; clomipramine, a FDA-approved drug for psychiatric disorders, which possesses an active metabolite (desmethylclomipramine) known to block autophagosome–lysosome fusion [112]; lucanthone, disrupts lysosomal membrane permeabilization [113]; Bafilomycin A1, inhibitor of the V-ATPase, which leads to the disruption of autolysosome acidification and to autophagosome-lysosome fusion [114]; Quinacrine, prevents the fusion of autophagosomes and lysosomes [108]; Quinacrine, Mefloquine, and GSK369796, inhibit the activity of the lysosomal cathepsin B presumably by direct binding to the enzyme [108]. 

**Figure 7:**
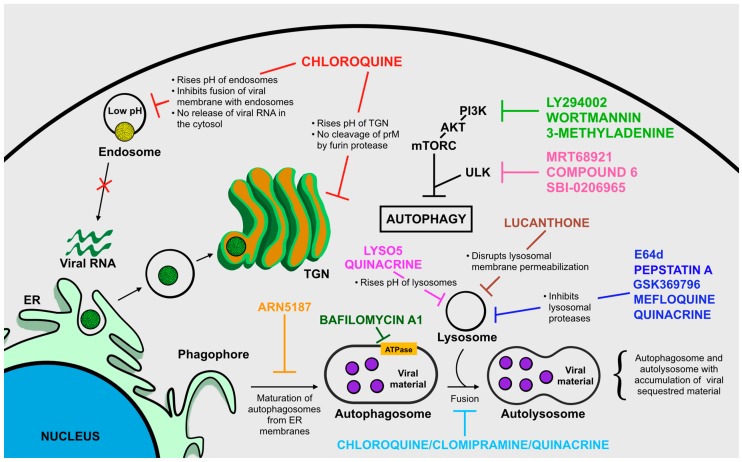
Compound testing and drug design to limit autophagy: a possible approach to counteract ZIKV infection. Different compounds possess the ability to modulate crucial steps for autophagy progression. Considering the fundamental role of autophagy in promoting viral replication and advancement of infection, the testing of molecules known to act on autophagy might be essential to identify and select candidates to test for anti-ZIKV activity.

As already indicated, the PI3K/Akt/mTORC signaling pathway is the main intracellular route involved in regulating the progression of autophagic flux in presence of various stress stimuli, including viral infections. We therefore suggest that testing well-established compounds known to alter the PI3K/Akt/mTORC axis should be executed to identify other potential drugs that could limit ZIKV infection. To support our hypothesis there has been a recent report demonstrating that pharmacological inhibition of autophagy using wortmannin—a microbial product and inhibitor of the early stages of autophagy by blocking PI3K—is associated to a reduction in ZIKV production in an in vitro model of human umbilical vein endothelial cells [50]. Other PI3K-blockers have been used as autophagy inhibitors such as: 3-methyladenine, widely used to inhibit autophagy in vitro [115,116]; and LY294002, the first synthetic inhibitor of PI3K (Figure 7) [115,117]. 

## 5. Conclusions

ZIKV infection still constitutes an ongoing challenge that requires strict surveillance and attention. There is the necessity to unravel ZIKV biology and the mechanisms involved in virus–host cell interactions in order to develop potential vaccines, therapies, and prophylactic regimes to counteract viral infection since they are not yet available. Recent findings support the assumption that autophagy might represent a potential target for therapeutic purposes. Therefore, understanding the specific molecular details of how ZIKV is able to engage autophagy will certainly clarify the roles of autophagic flux in viral replication and progression, and may help to assess effective therapeutics to potentially treat disease and control virus infection.
